# Rise in fasting and dynamic glucagon levels in children and adolescents with obesity is moderate in subjects with impaired fasting glucose but accentuated in subjects with impaired glucose tolerance or type 2 diabetes

**DOI:** 10.3389/fendo.2024.1368570

**Published:** 2024-07-04

**Authors:** Thomas Pixner, Tatsiana Chaikouskaya, Wanda Lauth, Georg Zimmermann, Katharina Mörwald, Julia Lischka, Dieter Furthner, Elisabeth Awender, Sabine Geiersberger, Katharina Maruszczak, Anders Forslund, Christian-Heinz Anderwald, Janne Cadamuro, Daniel Weghuber, Peter Bergsten

**Affiliations:** ^1^ Department of Pediatric and Adolescent Medicine, Salzkammergutklinikum Voecklabruck, Voecklabruck, Austria; ^2^ Obesity Research Unit, Paracelsus Medical University, Salzburg, Austria; ^3^ Institut national supérieur des sciences agronomiques de l'alimentation et de l'environnement, Dijon, France; ^4^ Department of Pediatrics, University Hospital Salzburg, Paracelsus Medical University, Salzburg, Austria; ^5^ Biostatistics and Big Medical Data, Lab for Intelligent Data Analytics (IDA) Salzburg, Paracelsus Medical University, Salzburg, Austria; ^6^ Clinical Research Center Salzburg, Paracelsus Medical University, Salzburg, Austria; ^7^ Department of Women’s and Children’s Health, Uppsala University, Uppsala, Sweden; ^8^ Division of Endocrinology and Metabolism, Department of Internal Medicine III, Medical University of Vienna, Vienna, Austria; ^9^ Direction, Arnoldstein Healthcare Centre, Arnoldstein, Austria; ^10^ Department of Laboratory Medicine, Paracelsus Medical University, Salzburg, Austria; ^11^ Department of Medical Cell Biology, Uppsala University, Uppsala, Sweden

**Keywords:** glucagon, OGTT, obesity, pediatric, liver-alpha cell axis, insulin, diabetes mellitus

## Abstract

**Background:**

Fasting levels of glucagon are known to be elevated in youth and adults with type 2 diabetes mellitus (T2D). Children and adolescents with obesity were previously reported to show increasing fasting and post-glucose-challenge hyperglucagonemia across the spectrum of glucose tolerance, while no data are available in those with impaired fasting glucose (IFG).

**Materials and methods:**

Individuals from the Beta-JUDO study population (Uppsala and Salzburg 2010–2016) (n=101, age 13.3 ± 2.8, m/f =50/51) were included (90 with overweight or obesity, 11 with normal weight). Standardized OGTT were performed and plasma glucose, glucagon and insulin concentrations assessed at baseline, 5, 10, 15, 30, 60, 90 and 120 minutes. Patients were grouped according to their glycemic state in six groups with normal glucose metabolism (NGM) and normal weight (NG-NW), NGM with obesity or overweight (NG-O), impaired glucose tolerance (IGT), impaired fasting glucose (IFG), IGT+IFG and T2D, and in two groups with NGM and impaired glucose metabolism (IGM), for statistical analysis.

**Results and conclusion:**

Glucagon concentrations were elevated in young normoglycemic individuals with overweight or obesity (NG-O) compared to normoglycemic individuals with normal weight. Glucagon levels, fasting and dynamic, increased with progressing glycemic deterioration, except in IFG, where levels were comparable to those in NG-O. All glycemic groups showed an overall suppression of glucagon during OGTT. An initial increase of glucagon could be observed in T2D. In T2D, glucagon showed a strong direct linear correlation with plasma glucose levels during OGTT. Glucagon in adolescents, as in adults, may play a role in the disease progression of T2D.

## 1 Introduction

The prevalence of pediatric obesity and its related comorbidities has constantly increased over the past decades (1, 2). Obesity is the main risk factor for type 2 diabetes mellitus (T2D) and the metabolic syndrome, sharing insulin resistance (IR) as a common feature (3, 4). The concept of diabetes mellitus has changed from a monohormornal, insulinocentric (lack of relative insulin quantity and sensitivity), to a bi- or even multihormonal disorder (5, 6). In recent years glucagon has gained increasing interest in both obesity and diabetes research. The glucagonocentric theory focuses on alpha cell dysfunction and is supported by studies on glucagon-receptor knock-out mice in which glycemic control was maintained by blocking glucagon action even in the relative absence of insulin. However, this effect is lost in complete lack of insulin (7–12).

Glucagon regulates blood glucose concentrations by stimulating hepatic glucose production (13). Blood glucose and insulin levels are considered essential regulators of glucagon (14). Under normal physiological conditions glucagon levels are expected to decline during an oral glucose tolerance test (OGTT) (15, 16). The exact mechanism of glucagon secretion is still incompletely understood and far more complex than previously considered. Currently proposed mechanisms of glucagon secretion include intrinsic (direct alpha cell modulation via glucose), endocrine, paracrine (inhibitory effects of glucose mediated via beta and delta cells), autocrine (self-stimulation) and neural, likely to function complementary (17, 18).

In adults with obesity and T2D, fasting hyperglucagonemia and a paradoxical increase as well as failure to suppress glucagon in response to an oral glucose challenge was shown (3, 14, 19). Similar phenotypic heterogeneity is also known to exist in nondiabetic adults living with obesity, contrary to lean individuals (20–24). The loss of beta cell function at a given IR is crucial in the development of T2D (25–27). In addition, fasting hyperglucagonemia appears early in the development of T2D (19, 28–31) and is acknowledged as a contributing factor (18, 27, 32, 33). In a 2021 study, both adults and youth (IGT and T2D only) displayed hyperglucagonemia, but glucagon concentrations were lower in youth. Additionally, an inverse relationship between fasting glucagon and fasting glucose in adolescents was observed (contrarily to adults). The authors concluded that alpha-cell dysfunction in youth does not appear to explain the observed beta-cell hyperresponsiveness and insulin-resistance (34) while elevation of glucagon (fasting and postprandial) has been suggested to drive hyperglycemia in other studies (27, 35, 36).

In children and adolescents with obesity and NGT, IGT or T2D, we observed that fasting hyperglucagonemia increased with degree of glycemic derangement (37). Also, increase of circulating glucagon during the first 15 min of OGTT and less suppression of glucagon levels during the glucose load was seen, especially in subjects with T2D. In youth with obesity and IGT or T2D fasting glucose and glucagon were negatively correlated and hyperglucagonemia does not appear to drive hyperglycemia (34).

In adults with impaired fasting glucose (IFG) with or without impaired glucose tolerance (IGT), higher plasma glucose concentrations were necessary to suppress glucagon secretion compared to individuals with normal glucose metabolism (NGM) (38). This implies that alpha cell insensitivity contributes to IFG rather than glucose tolerance (38). No previous pediatric studies on glucagon dynamics in subjects with IFG (or IGT+IFG) exist. This and the question if adult and pediatric data are comparable imply the need to extend previous studies and include IFG when investigating glycemic states.

In the present study we have built on our previous work (37) and focused on subjects with IFG and IGT+IFG. The aim is to investigate fasting and dynamic glucagon levels during an OGTT in children and adolescents with IFG and compare them with subjects with differing glycemic state.

## 2 Materials and methods

### 2.1 Study population and design

All individuals included in this study were volunteering in the BETA JUDO study (Beta-cell function in Juvenile Diabetes and Obesity, FP7-HEALTH-2011-two stage, project number: 279153). Study subjects, aged 10 to 18 years, with overweight or obesity (BMI-SDS >1.26) were recruited at Uppsala University Hospital, Sweden and at Paracelsus Medical University Hospital in Salzburg, Austria. Exclusion criteria were type 1 diabetes mellitus, syndromic obesity, psychiatric disorders, allergies, alcohol intake, presence of chronic liver disease and/or hereditary liver disease, consummation of steatogenic substances and endocrine disorders. Ethics approval was obtained from both Uppsala regional ethical committee (Number 2012/318) and the Salzburg ethical committee (Number 1544/2012). The study followed the Declaration of Helsinki and written informed consent was obtained beforehand from every participant and one or both caregivers. This retrospective cross-sectional study uses material and data obtained in the course of the BETA-JUDO study.

### 2.2 Oral glucose tolerance test, blood sampling, and biochemical measurements

Participants fasted overnight and then underwent a standard oral glucose tolerance test (OGTT), as previously described (39). Individuals received a glucose solution (1,75 g glucose/kg, 75g glucose maximum) and blood samples were taken via a venous catheter at -5 (in the text also referred to as fasting), 5, 10, 15, 30, 60, 90, 120 min. Plasma was separated by immediate centrifugation of blood samples (2500g for 10 minutes at 4°C) and frozen (-80°C). Glucose in Uppsala was analyzed via an Architect c8000 instrument (Abbott Diagnostics, Solna, Sweden) and in Salzburg using a Gluco-Quant Glucose-Kit (Roche Diagnostics, Mannheim, Germany). Laboratories in both clinical centers performed validation of their glucose analyses using reference blood samples. Plasma insulin and glucagon was analyzed in Uppsala for both Uppsala and Salzburg, using monoclonal based sandwich ELISAs (Mercodia AB, Uppsala, Sweden). Assay detection levels for insulin were 6.9 pmol/L (no cross reactivity with c-peptide or proinsulin). For glucagon detection level was 1 pmol/L. Interassay variability was controlled via standardized control samples (Mercodia AB, Uppsala, Sweden).

### 2.3 Anthropometric assessment, magnetic resonance imaging, and grouping by glycemic state

Measurements of height and weight were assessed via standardized and calibrated stadiometers (Ulmer (Busse Design and Engineering GmbH; Eichingen, Germany) in Uppsala and Seca (Seca, Hamburg, Germany) in Salzburg). Age- and sex-adjusted Body mass index (BMI) was calculated in accordance with WHO growth report and using Microsoft Excel add-in LMS Growth. Individuals were grouped with overweight or obesity if BMI-SDS was >1.26 (40). Waist circumference was measured at the umbilical level and recorded in centimeters. Magnetic resonance image scans (MRI) to obtain liver fat content were performed, using 1,5 Tesla clinical MRI by Philips Medical System (Netherlands), according to a previously reported method (39). Grouping for glycemic state was performed according to WHO criteria and ISPAD (International Society for Pediatric and Adolescent Diabetes). Normal fasting glucose (NFG) is defined as less than 100 mg/dl (5.6 mmol/L). Impaired fasting glucose (IFG or prediabetes) per definition is 100–125 mg/dl (5.6–6.9) serum glucose, while levels of ≥126 mg/dl (≥7.0 mmol/L) is defined as type 2 diabetes mellitus (T2D). The 120 minute-value of the OGTT defines <140 mg/dl (<7.8 mmol/L) as normal glucose tolerance (NGT), levels of 140–199 mg/dl (7.8–11.0 mmol/L) as impaired glucose tolerance (IGT) and ≥200 mg/dl (11.1 mmol/L). If individuals fulfilled any one of the T2D criteria they were categorized as T2D. If they met the criteria for IGT and IFG they were grouped as IGT+IFG (41, 42).

### 2.4 Estimation of hepatic insulin resistance

For the estimation of the hepatic insulin resistance the homeostatic model assessment of insulin resistance (HOMA-IR) was used. HOMA-IR is calculated as product of mean fasting glucose (in mmol/L) and fasting insulin (in µU/mL) which is then divided by the constant 22.5 (43).

### 2.5 Statistical analysis

For data analysis, the characteristics as well as the outcome variables of six subgroups (NGM (normal glucose metabolism); NG-NW (normal glucose metabolism with normal weight); NG-O (normal glucose metabolism with overweight or obesity); IGM (impaired glucose metabolism); IFG, IGT+IFG (individuals fulfilling criteria of both IGT and IFG), T2D) were first considered descriptively by calculating means, standard deviations and absolute frequencies. Patients with normal (NG-NW, NG-O) and abnormal glucose tolerance (IFG, IGT, IGT+IFG and T2D) were combined into normal glucose metabolism (NGM) and impaired glucose metabolism (IGM), respectively. In addition, differences between the two groups in metric values were examined by using a non-parametric approach using the Wilcoxon test and for the ordinal values using the Fisher exact test. Because of the large number of related tests, Bonferroni-Holm was used to adjust for multiplicity. To detect an increase in glucagon levels compared to baseline (fasting), they were considered relatively regarding the baseline (fasting) values. To account for correlations between glucose and glucagon levels, the Pearson correlation coefficient per time point was considered for both two and all six groups. A correlation coefficient of <0.2 was defined as no correlation, 0.2–0.5 as weak, 0.5–0.8 as clear/moderate and >0.8 as high/strong correlation. The AUC (area under insulin and glucagon curve for the six groups) was calculated using the trapezoid method. Two-sided significance level of α = 0.05 was used. All analyses were carried out using the statistical software R (44).

## 3 Results

### 3.1 Baseline characteristics

The clinical and anthropometric features of the study cohort are shown in **Table 1**. One hundred and one individuals were included with a mean age of 13.38 ± 2.81 years and a mean BMI-SDS of 2.55 ± 1.43. Ninety individuals were classified as living with overweight or obesity. Eleven adolescents had normal weight and normal fasting glucose and tolerance. Liver fat fraction data was available in a subgroup of 18 subjects with a mean of 4.78 ± 4.82%. Liver fat fraction was highest in T2D, followed by IGT+IFG, NGM (normal glucose metabolism) with obesity (NG-O), IGT, IFG and NGM with normal weight (NG-NW). HOMA-IR was available in 99 patients with a mean of 4.55 ± 4.33. HOMA-IR was highest in T2D, followed by IGT+IFG, IFG, IGT, NG-O and NG-NW.

**Table 1:** Baseline characteristics.

	Total	NGM	NG-NW	NG-O	IGM	IFG	IGT	IGT+IFG	T2D
**Participants**	n=101	n=40	n=11	n=29	n=61	n=23	n=12	n=22	n=4
**Male/Female**	50/51	15/25	4/7	11/18	35/26	15/8	5/7	14/8	1/3
**Age (years)**	13.3 ± 2.8	12.8 ± 2.7	13.3 ± 2.2	12.6 ± 2.9	13.7 ± 2.8	13.2 ± 2.6	12.7 ± 3.66	14.6 ± 2.5	14.6 ± 2.3
**Height (cm)**	162 ± 14.4	160.5 ± 14.6	160 ± 11.7	160.6 ± 15.7	164 ± 14.3	163.8 ± 16.2	155 ± 12.6	169 ± 12.1	164 ± 8.2
**Waist (cm)**	97 ± 20	91.9 ± 21.1	67.7 ± 5.5	103 ± 15.4	101.3 ± 20	86.5 ± 18.9	99.8 ± 9.8	117.1 ± 13.2	116 ± 12.8
**Weight (kg)**	85 ± 32.8	77,2 ± 32.5	47.8 ± 11.0	88.4 ± 31	90.3 ± 32.2	77 ± 31	79.2 ± 30.1	107.9 ± 27.1	103 ± 28
**BMI (kg/m^2^)**	31 ± 8.4	29.1 ± 9.01	18.4 ± 2.4	33.1 ± 7	32.5 ± 7.9	27.7 ± 7.3	31.9 ± 7.2	37.1 ± 5.5	37.9 ± 8.3
**BMI-SDS**	2.5 ± 1.4	2.1 ± 1.6	-0.26 ± 0.89	3.1 ± 0.42	2.7 ± 1.24	2.0 ± 1.68	3.1 ± 0.55	3.3 ± 0.3	3.3 ± 0.6
**HOMA-IR**	4.5 ± 4.3	2.9 ± 1.8	1.2 ± 0.3	3.6 ± 1.7	5.7 ± 5.1	3.7 ± 1.9	4.08 ± 2.0	6.0 ± 3.3	19.4 ± 10.0
**HbA1c (mmol/mol) (%)**	35 ± 3.2 5.4 ± 0.3	34.2 ± 3.0 5.3 ± 0.3	33.5 ± 1.6 5.2 ± 0.2	34.4 ± 3.3 5.3 ± 0.3	35.5 ± 3.3 5.4 ± 0.3	34.4 ± 2.8 5.3 ± 0.3	35 ± 3.2 5.4 ± 0.3	36.2 ± 2.8 5.5 ± 0.3	39 ± 5.0 5.8 ± 0.5
**Liver-Fat Fraction (%)**	4.7 ± 4.8	3.4 ± 3.5	1.2 ± 0.4	6.6 ± 3.7	5.6 ± 5.8	4.5 ± n.a.	2.6 ± 0.2	9.7 ± 7.3	14 ± n.a.

BMI (body mass index); BMI-SDS (body mass index – standard deviation score); HOMA-IR (homeostatic model assessment of insulin resistance); NGM (normal glucose metabolism); NG-NW (normal glucose metabolism with normal weight); NG-O (normal glucose metabolism with overweight or obesity); IGM (impaired glucose metabolism); IFG (impaired fasting glucose); IGT (impaired glucose tolerance); IFG+IGT (group fulfills criteria for IGT and IFG); T2D (Type 2 Diabetes mellitus). n.a., not applicable.

### 3.2 Glucagon in normoglycemic individuals with normal weight and obesity

**Figure 1:**
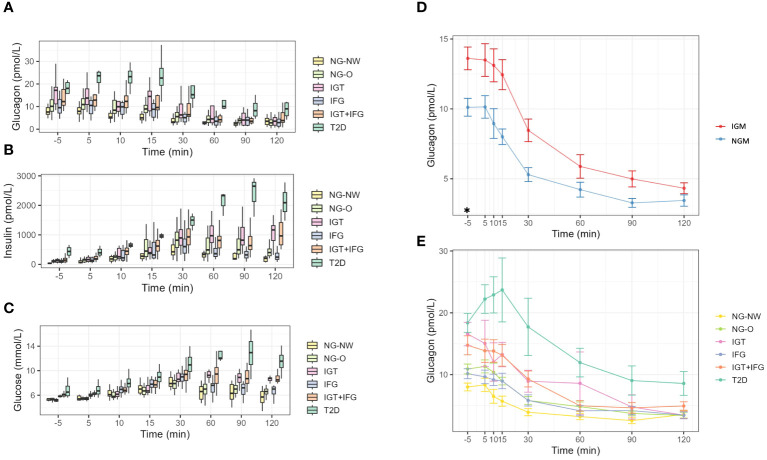
Dynamics during OGTT. **(A)** Glucagon dynamics in the six groups. **(B)** Insulin dynamics in the six groups. **(C)** Glucose dynamics in the six groups. **(D)** Line-graphs displaying mean glucagon dynamics of NG and IGM during OGTT. **(E)** Line-graphs displaying mean glucagon dynamics during OGTT of the 6 subgroups. NGM (normal glucose metabolism); NG-NW (normal glucose metabolism with normal weight); NG-O (normal glucose metabolism with overweight or obesity); IGM (impaired glucose metabolism); IFG (impaired fasting glucose); IGT (impaired glucose tolerance); IFG+IGT (group fulfills criteria for IGT and IFG); T2D (Type 2 Diabetes mellitus); *p<0.05.

The glucagon results of the respective groups are presented in **Table 2**, **Figure 1**. Mean fasting glucagon in normoglycemic individuals (NGM) was significantly lower (p<0.001; p<0.05 after adjusting for age, HbA1c and fasting insulin) by 25.7% than in those with impaired glucose metabolism (IGM) (10.12 ± 4.03 vs. 13.61 ± 6.34 pmol/L, respectively). Fasting glucagon range widely varied between groups with NGM 4.0–23.39 vs. NG-NW 4.8–11.3 vs. NG-O 4.0–23.39 pmol/L). The NG-O group displayed higher mean fasting glucagon concentrations than the NG-NW group by 26% (10.91 ± 4.32 vs. 8.01 ± 2.07 pmol/L, respectively). Dynamic glucagon levels were significantly lower in NGM compared to IGM during the first 30 minutes of OGTT. This effect was lost after adjusting for age, HbA1c and fasting insulin. Throughout OGTT glucagon levels in NG subjects never exceeded those in IGM subjects, respectively. In NGM subjects glucagon concentrations remained sTable within the first 5 minutes and constantly decreased thereafter. The initial increase of glucagon_5min_ levels was slightly more pronounced in NG-O (10.91 ± 4.32 to 11.33 ± 4.05 pmol/L) than in NG-NW (8.01 ± 2.07 to 8.35 ± 3.38 pmol/L). At the end of OGTT glucagon_120min_ was below baseline in NGM subjects, NG-NW and NG-O (-64.6%, -55.3% and -68.9% respectively). Only one individual, belonging to the NG-NW group, displayed glucagon_120min_ above baseline (**Table 2**, **Figure 1**). Mean glucagon levels in NG-O were 26.3% to 36.2% higher compared to NG-NW between minute 5 and 90. In contrast, mean glucagon_120min_ was 5.6% higher in NG-NW than in NG-O. Investigation of mean ΔGlucagon (Glucagon_120_ - Glucagon_fasting_) resulted in NG-NW being closer to baseline (-4.43 ± 2.98 pmol/L), than NG-O (-7.52 ± 4.01 pmol/L).

**Table 2:** Mean±Standard deviation values for glucagon, insulin and glucose during OGTT.

		Total	NGM	NG-NW	NG-O	IGM	IFG	IGT	IGT+IFG	T2D	p-value	p-value adjusted
Glucagon (pmol/L)												
Fasting		12.23±5.78	10.12±4.03	8.01±2.07	10.91±4.32	13.61±6.34	10.16±3.89	16.57±6.56	14.75±7.18	18.38±3.08	<0.05*	<0.05*
5		12.07±5.99	10.14±4.01	8.35±3.38	11.33±4.05	13.5±6.82	9.64±3.79	15.05±7.5	13.86±7.07	22.23±4.61	<0.05*	0.57371
10		11.33±6.73	8.95±5.52	6.49±3.53	10.4±6.05	13.11±7.07	9.16±3.04	12.05±7.58	13.83±7.06	22.9±5.85	<0.05*	0.09697
15		10.69±6.7	8.01±3.24	5.71±2.57	8.96±3.04	12.45±7.75	8.98±5.45	13.26±5.92	13.13±7.86	23.7±10.34	<0.05*	0.12187
30		7.22±5.45	5.29±3.11	3.93±1.9	5.83±3.35	8.46±6.26	5.84±4.35	8.94±6.13	9.31±5.92	17.73±9.27	<0.05*	0.14666
60		5.21±4.22	4.22±2.6	3.22±1.47	4.82±2.98	5.88±4.97	4.16±2.99	8.6±10.15	4.96±3.18	11.95±4.63	0.2765	0.79498
90		4.31±2.91	3.27±1.53	2.57±1.42	3.73±1.47	4.99±3.39	4.18±2.4	4.77±3.94	4.64±3.19	9.05±4.73	<0.01*	0.66268
120		3.97±2.87	3.44±2.56	3.58±1.94	3.39±2.78	4.32±3.03	3.45±2.51	3.45±2.01	4.93±3.2	8.57±3.85	0.16487	0.79498
Insulin (pmol/L)												
Fasting		122.32±98.26	87.52±53.34	36.24±9.74	106.97±50.03	146.32±114.2	99.5±48.51	123.21±61.74	153.55±85.75	436.92±199.38	<0.05*	<0.05*
5		177.12±130.07	143.81±131.7	93.02±69.34	165.9±146.99	201.02±124.86	172.29±124.92	174.55±90.83	204.69±98.85	420.87±198.03	<0.05*	0.09876
10		351.78±230.15	269.88±199.7	215.03±157.93	296±215.35	406.98±234.87	338.63±264.21	353.98±199.53	458.42±218.22	651.5±96.28	<0.05*	0.16436
15		549.83±343.86	487.74±348.56	356.93±267.92	556.59±372.23	594.84±337.62	523.01±412.31	460.28±215.57	651.65±305.02	962±93.42	0.14375	0.79498
30		853.56±490.36	750.73±476.6	541.65±372.96	850.29±495.92	922.85±492.3	707.06±464.81	960.11±436.37	1012.89±506.72	1456.97±289.3	0.11357	0.79498
60		744.86±558.91	618.27±544.04	316.64±113.76	749.42±604.88	833.75±557.6	487.56±357.73	1026.75±491.6	807.92±382.21	2118.8±412.55	<0.05*	0.46804
90		717.29±594.43	577.21±497.73	289.28±185.38	702.4±540.61	815.65±640.7	385.21±252.85	1016.74±490.96	1016.74±490.96	2393.77±688.63	<0.05*	0.47661
120		695.26±607.81	380.33±273.8	181.94±87.37	479.53±282.39	911.78±678.4	307.32±212.67	1112.65±506.09	1125.45±598.18	2117.03±647.63	<0.001*	<0.01*
Glucose (mmol/L)												
Fasting		5.64±0.57	5.28±0.21	5.25±0.25	5.28±0.2	5.88±0.6	5.87±0.24	5.18±0.2	6.1±0.33	6.88±1.47	<0.001*	<0.001*
5		5.86±0.64	5.53±0.56	5.51±0.38	5.54±0.61	6.08±0.6	6.09±0.44	5.5±0.37	6.23±0.42	6.9±1.27	<0.001*	<0.001*
10		6.58±0.94	6.2±0.94	6.5±1.6	6.09±0.79	6.84±0.85	6.86±0.72	6.23±0.62	6.91±0.62	8.1±1.66	<0.001*	<0.001*
15		7.43±1.09	6.93±1.05	7.27±1.3	6.79±0.91	7.76±0.99	7.84±0.85	7.02±0.92	7.8±0.75	9±1.7	<0.001*	<0.01*
30		8.65±1.41	7.85±0.9	8.19±0.99	7.72±0.85	9.18±1.44	8.83±1.15	8.79±0.88	9.4±1.55	11.12±2.28	<0.001*	<0.001*
60		8.09±2.15	6.95±1.52	6.52±1.29	7.11±1.59	8.83±2.19	7.35±1.37	9.32±1.27	9.36±1.99	13.02±2.26	<0.001*	<0.001*
90		7.79±1.88	6.88±1.14	6.38±1.47	7.07±0.95	8.39±2.04	7.03±1.26	8.72±1.13	8.83±1.37	12.8±3.44	<0.001*	<0.001*
120		7.4±1.62	6.29±0.93	5.63±1.27	6.54±0.64	8.13±1.57	6.67±0.81	8.72±0.54	8.72±0.67	11.47±2.24	<0.001*	<0.001*

NG (normal glucose metabolism); NG-NW (normal glucose metabolism with normal weight); NG-O (normal glucose metabolism with overweight or obesity); IGM (impaired glucose metabolism); IFG (impaired fasting glucose); IGT (impaired glucose tolerance); IFG+IGT (group fulfills criteria for IGT and IFG); T2D (type 2 diabetes mellitus); p-values for comparing levels between NGM and IGM; p-values adjusted for age, HbA1c and fasting insulin. *result of statistical significance.

Fasting insulin levels differed clearly between NG-NW and NG-O (36.24 ± 9.74 vs. 106.97 ± 50.03). Mean insulin levels peaked in both groups (NG-NW and NG-O) at 30 minutes, to then decline. NG-O mean insulin levels were 35.8–66.1% higher in the first 30 minutes than those in NG-NW and 57.7–62% thereafter. Fasting concentrations for serum glucose were almost equal in NG-NW, NG-O (5.25 ± 0.25vs. 5.28 ± 0.2 mmol/l). In NG-NW and NG-O serum glucose levels constantly rose until 30 minutes, to then decline. Please see **Table 2**, **Figure 1** for details.

### 3.3 Glucagon in individuals with impaired glucose metabolism

All patients with impaired glucose metabolism (i.e. IFG, IGT, IGT+IFG and T2D) were classified as overweight or obese. Due to the small sample sizes, especially in the T2D group, only descriptive statistical analyses are presented (**Table 2**, **Figure 1**). Fasting glucagon in IGM subjects was significantly higher than in NGM subjects (13.61 ± 6.34 vs. 10.12 ± 4.03 pmol/L, p<0.05). Mean baseline glucagon differed between the four groups (IFG 10.16 ± 3.89 vs. IGT 16.57 ± 6.56 vs. IGT+IFG 14.75 ± 7.18 vs. T2D 18.38 ± 3.08 pmol/L) as did fasting glucagon range (IGT 8.4–28.97 vs. IFG 4.6–17.06 vs. IGT+IFG 8–36.98 vs. T2D 15.7–21.7 pmol/L). Mean fasting glucagon in T2D was 129% higher than in NG-NW. During OGTT, groups displayed an overall decrease of plasma glucagon levels with intermittent increases. In IGM subjects mean glucagon levels were higher throughout OGTT compared to subjects with NGM (**Table 2**). While fasting glucagon levels of individuals with IGT were higher than those with IFG. From minute 10 to 15 there was a slight increase in mean glucagon in the IGT group (from 12.05 ± 7.58 to 13.26 ± 5.92 pmol/L). In the IFG and the IGT+IFG groups there was a constant decline of mean glucagon concentrations.

In patients with T2D there was a pronounced initial increase in plasma glucagon within the first 30 minutes of the OGTT (glucagon_fasting_ 18.38 ± 3.08, glucagon_5min_ 22.23 ± 4.61, glucagon_10min_ 22.90 ± 5.85, glucagon_15min_ 23.70 ± 10.34 pmol/L). At 30 minutes glucagon was below baseline to consistently decline until the end of measurement. Mean glucagon_120min_ in IGT was similar to IFG and NGM groups (IGT 3.45 ± 2.01 vs. IFG 3.45 ± 2.51 vs. NG-NW 3.58 ± 1.94 vs. NG-O 3.39 ± 2.78 pmol/L), while in IGT+IFG and T2D levels were distinguishable higher (IGT+IFG 4.93 ± 3.2 vs. T2D 8.57 ± 3.85 pmol/L). Mean ΔGlucagon (Glucagon_120_ - Glucagon_fasting_) did not follow deterioration of glycemic state (IFG -6.71 ± 3.42 vs. T2D -9.8 ± 1.2 vs. IGT+IFG -9.82 ± 5.74 vs. IGT -13.12 ± 6.0 pmol/L).

Fasting insulin levels also differed clearly (IFG 99.5 ± 48.51vs. IGT 123.21 ± 61.74 vs. vs. IGT+IFG 153.55 ± 85.75 vs. T2D 436.92 ± 199.38 pmol/L). In the IFG group maximum insulin concentrations were observed at 30 minutes. In the IGT+IFG group two insulin peaks were noticeable (30 and 120 minutes). Insulin levels initially dropped in the T2D group, constantly increasing thereafter from 5 minutes until 90 minutes to then decrease. T2D insulin levels remained the highest of all groups throughout OGTT. Insulin levels were significantly higher in the IGM group at fasting. Please see **Table 2** for mean-values and SD. Fasting concentrations for mean plasma glucose in IGT were comparable to NG-NW and NG-O (5.18 ± 0.2 vs. 5.25 ± 0.25 vs. 5.28 ± 0.2 mmol/L, respectively) and higher in IFG, IGT+IFG and T2D (5.87 ± 0.24 vs. 6.1 ± 0.33 vs. 6.88 ± 1.47 mmol/L respectively). In NG-NW and NG-O serum glucose levels constantly rose until 30 minutes, to then decline. The turning-point for glucose-dynamics was at 30 minutes for IFG and IGT+IFG and at 60 minutes for IGT and T2D. Insulin levels were significantly higher in the IGM(all) group at fasting. Please see **Table 2**, **Figure 1** for details.

### 3.4 AUC for glucagon and insulin

The AUC (area under the curve) for glucagon and insulin was also calculated (**Table 3**.). The lowest AUC (mean) for glucagon was found in NG-NW (436.60 pmol/L), followed by IFG (547.62 pmol/L), NG-O (610.27 pmol/L), IGT+IFG (749.52 pmol/L), IGT (979.25 pmol/L) and T2D (1564.50 pmol/L). AUC (mean) insulin was lowest in NG-NW (38.14 nmol/L), followed by IFG (54.79 nmol/L), NG-O (79.17 pmol/L), IGT+IFG (96.19 nmol/L), IGT (101.06 nmol/L), and T2D (213.84 nmol/L). The order of AUCs for glucagon and insulin (low to high) followed the groups glycemic status, except for IFG.

**Table 3:** Area under the curve (AUC) for glucagon and insulin (Mean ± SD).

	NG-NW	NG-O	IFG	IGT	IGT+IFG	T2D
**AUC Glucagon** **(pmol/L)**	436.60 ± 154.50	610.27 ± 232.0	547.62 ± 267.16	979.25 ± 908.11	749.52 ± 460.8	1564.5 ± 664.42
**AUC Insulin** **(nmol/L)**	38.14 ± 19.66	79.17 ± 52.80	54.79 ± 30.04	101.06 ± 49.59	96.19 ± 41.69	213.84 ± 25.54

NG-NW (normal glucose metabolism with normal weight); NG-O (normal glucose metabolism with overweight or obesity); IFG (impaired fasting glucose); IGT (impaired glucose tolerance); IFG+IGT (group fulfills criteria for IGT and IFG); T2D (Type 2 Diabetes mellitus).

### 3.5 Correlation of glucagon and plasma glucose

**Figure 2:**
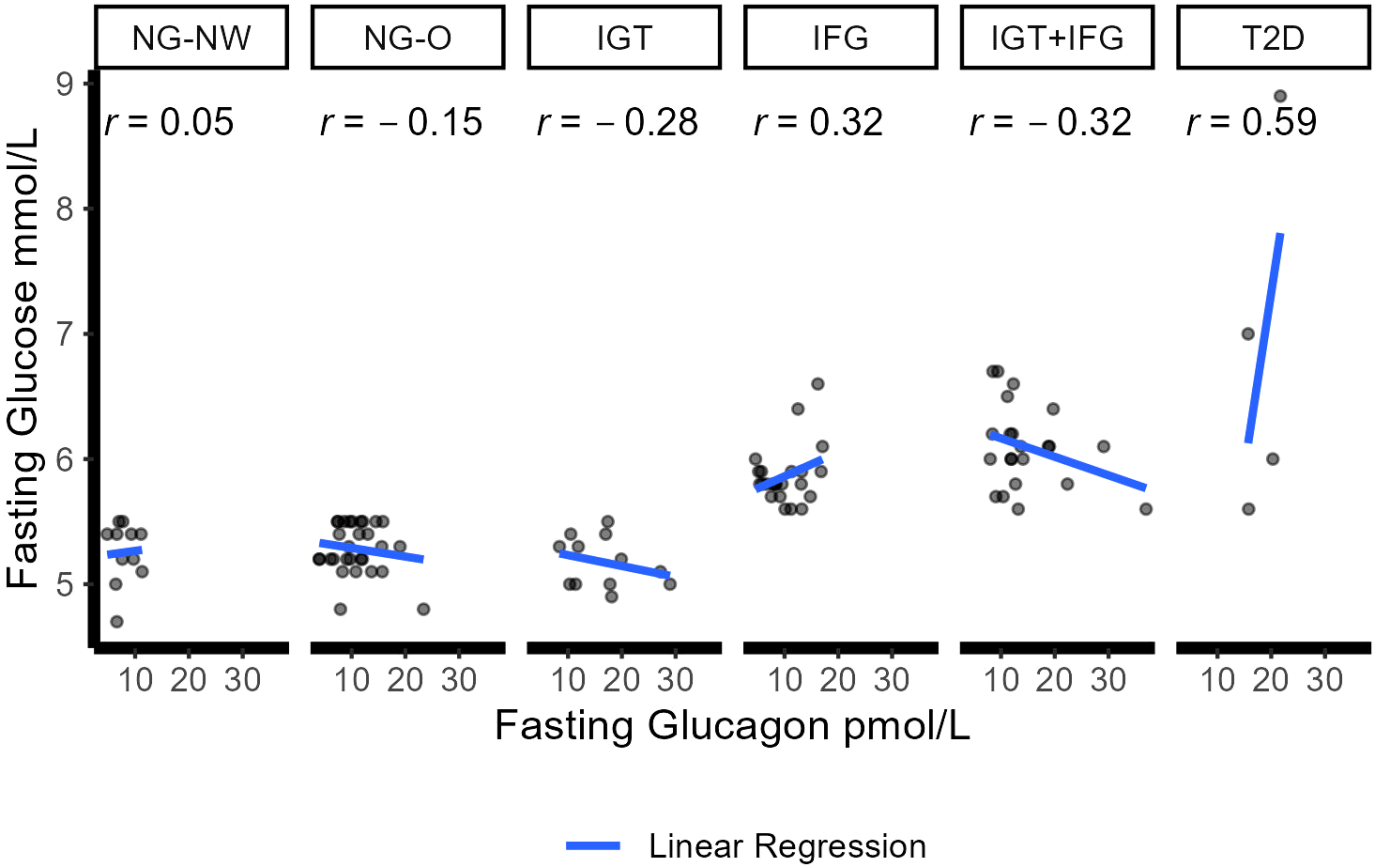
Correlation between fasting glucose and glucagon in the six groups. Dots represent individual correlations. r represents the Pearson correlation coefficient for each group (<0.2 no correlation; 0.2–0.5 weak correlation; 0.5–0.8 clear/moderate correlation; >0.8 high/strong correlation. Blue line displays linear regression between glucose and glucagon. NGM (normal glucose metabolism); NG-NW (normal glucose metabolism with normal weight); NG-O (normal glucose metabolism with overweight or obesity); IGM (impaired glucose metabolism); IFG (impaired fasting glucose); IGT (impaired glucose tolerance); IFG+IGT (group fulfills criteria for IGT and IFG); T2D (Type 2 Diabetes mellitus).

Fasting glucose showed a weak positive correlation was found in the IFG group (Pearson Correlation coefficient of r=0.32) and a clear one in the T2D cohort (r=0.59). A weak negative relationship in the IGT and the IGT+IFG groups (r=-0.28 and r=-0.32 respectively). No linear relationships were present in the NG-NW (0.04) and the NG-O (r=-0.14) groups. **Figure 2** illustrates the correlation between fasting and glucagon for the six different groups. From 5 minutes to 90 minutes no linear relationship was observed in NG-O. In individuals with NG-NW no linear relationship, except for minute 30 (r=0.29), was detected until minute 90 and 120 (r=-0.8). In IFG, a weak relationship was present from minute 15 until the end of OGTT. In IGT a weak linear correlations could be detected except for minute 60 (r=-0.69). In the T2D group a clear linear relationship could be noted during early glucagon increase at 5 minutes (r=0.77), as well as at 90 (r=0.59) and 120 minutes (r=0.70). Very strong correlations were detected in the middle of the OGTT,i.e.at 10, 15, 30 and 60 minutes (r_10_ = 0.98,r_15_ = 0.97,r_30_ = 0.95, r_60_ = 0.98).

## 4 Discussion

In our cohort of Austrian and Swedish youth with overweight and obesity (and normal weight controls), we were able to thoroughly investigate glucagon in regard to different glycemic states, ranging from normal glucose metabolism to prediabetes (distinguishing between IGT and IFT) and T2D. In our previous work (based on the Uppsala Longitudinal Study of Childhood Obesity) we investigated alpha-cell response during OGTT in NGT, IGT and T2D controls (37). The current study provides a larger cohort and corroborates previous findings but also provides more detailed information on glucagon in IFG as well as IGT+IFG, i.e. for the first time including all glycemic states. Although we find a weak correlation between fasting glucagon and glucose levels in subjects with IFG, glucagon concentrations are not elevated in subjects with IFG, as is the case in subjects with IGT (IGT, IGT+IFG, T2D), compared to normoglycemic individuals with overweight or obesity. Fasting and dynamic glucagon levels are, however, higher than in normoglycemic individuals with normal weight.

In the prediabetic groups fasting and dynamic glucagon levels were lowest in IFG, highest in IGT individuals and in between for IGT+IFT. Mean glucagon concentrations for IFG were below those of NG-O until minute_30_ and above thereafter. Contrary to prior results, no early increase in glucagon was observed in patients with IGT in this cohort (37, 45). The comparable low glucagon concentrations in IFG subjects were unexpected as increasing hyperglucagonemia in the progression of glycemic deterioration has been reported (1, 10). As none of the previous pediatric papers on glucagon dynamics included an IFG group there are no prior results to compare with (34, 37, 46, 47). In adults, lower glucagon in IFG than in IGT, IGT+IFG and T2D (T2D been the highest) has been reported (48). It is noteworthy that in adults Unger et al. have described how hyperglycemia in healthy individuals results in a suppression of glucagon to subnormal levels, while this effect is dramatically attenuated in individuals with diabetes mellitus (49). This effect likely translates from T2D to IFG, resulting in the combination of relatively too high glucagon levels and too low insulin levels for the prevailing glycemia, respectively. Hepatic insulin resistance was proposed to be a main characteristic of IFG, while peripheral insulin resistance is a dominant feature of IGT. In individuals with IGT+IFG it is likely that different pathophysiological mechanisms are at play (50–52). In our cohort, HOMA-IR was lower in IFG compared to IGT. The use of insulin and glucagon modeling may provide additional insight. In adult individuals with NFG/NGT and those with IFG insulin action (i.e. insulin sensitivity and responsiveness) (31) and insulin-stimulated glucose disposal did not differ but were reduced in IGT, IGT+IFG and patients with diabetes (48). Faerch et al. postulated that impaired insulin action (but not insulin secretion) is associated with higher glucagon concentrations (fasting and 30-min postprandial) (21). This in line with data implying that in normoglycemic adult individuals, alpha cell function is modulated (directly or indirectly) by the prevailing level of insulin action and in IGT impaired glucagon suppression correlated with decreased insulin action (53, 54). Although the aforementioned articles (21, 52, 53) use different estimates of insulin sensitivity to draw their conclusions they point towards the role of insulin action. Therefore, higher insulin action in pediatric IFG, compared to other forms of prediabetes, may additionally explain the relatively lower glucagon levels placing them in the range of NG-O. In our cohort of individuals with IGM, those with IFG display a lower BMI, lower HbA1c and better glucose control than in IGT, IFG+IGT and T2D. We deduce that insulin action is maintained as also AUC of insulin is lower (see **Table 3**) and insulin and glucagon kinetics are better controlled than in IGT and T2D (see **Figure 1**).

Furthermore, fasting glucose by itself has been described as a poor marker of beta-cell dysfunction in adult individuals with IFG (55). Kohlenberg et al. used the oral minimal model, investigating beta cell response to static and dynamic glucose concentrations, and found that both were normal in IFG, while this was not the case in IGT. They reported glucagon secretion rate (GSR) displayed a single-exponential relationship with glucose. Glucose necessary to suppress GSR was higher in IFG+IGT and IFG compared to IGT and NGM, suggesting that alpha cell insensitivity to glucose contributes to development of IFG (38). In adults fasting hyperglucagonemia has been linked to subsequent beta cell dysfunction and progression to T2D (56). Prediabetic alpha cell dysfunction appears to occur early and independent of beta cell dysfunction manifesting as fasting hyperglycemia (56, 57). Fasting hyperglucagonemia is considered a predictor of IGT (37, 38, 58), has been attributed to chronic hypersecretion and impaired suppression in hyperglycemia (31, 59) and to induce or worsen hyperglycemia (27). Results for elevated glucagon secretion during OGTT or meal consumption and T2D progression remain contradicting (6, 14, 56).

We found fasting glucagon and dynamic glucagon concentrations to be elevated in NG-O compared to NG-NW, paralleling previous pediatric data including ours (37, 47, 58). In a previous study we found higher fasting glucagon in individuals with obesity compared to controls with normal weight to be significantly associated with higher insulin, free fatty acids, triglycerides and visceral adipose tissue (VAT) (47). The association between VAT, excess FFAs and triglycerides is established and lipids have been shown to stimulate glucagon secretion (60–62). A recent pediatric study demonstrated glucagon secretion to also depend on insulin sensitivity. Adolescents with obesity and insulin resistance displayed elevated fasting glucagon levels and tAUC_0-120_ for glucagon, compared to insulin-sensitive individuals with obesity or normal weight (46).

Fasting and dynamic glucagon levels were highest in T2D (although this subgroup consisted of only 4 individuals). This and failure to suppress glucagon during OGTT were previously reported by our group (37). This is in line with adult studies (21, 28) including recent glucagon modeling data that demonstrated reduced suppression and increased secretion in T2D (63).

In this study cohort a stronger late suppression of glucagon throughout the OGTT for T2D, IGT and IGT+IFG was noTable. Similar dynamics were found in adults with prediabetes and T2D (21). In a previous study we could document that higher fasting insulin was associated with less glucagon suppression in the early phase of OGTT, whereas relative suppression at min_120_ did not differ based on fasting insulin (47). Results from adult glucagon modeling studies found glucagon suppresion to be negatively and linearly correlated with HbA1c, fasting glucose and 2 hour plasma glucose (63). In adult studies, increasing glucagon (i.e. above baseline) at the end of OGTT (i.e. 120 minutes) appears favorable as individuals were leaner, fasting glucagon and glucose levels were lower, insulin sensitivity higher and IGT risk was lower (20, 64). In contrast to these adult studies, only a single subject in our study cohort, belonging to the NG-NW group, displayed a greater glucagon_120min_ concentration above baseline.

In the pediatric population, there is only limited data on dynamic changes of glucagon during OGTT and no pediatric glucagon modeling studies exist yet. Previous pediatric studies were performed with fewer participants and subgroups (e.g. NGM vs. IGM, no IFG or IGT+IFG groups) (34, 37, 46, 47). While there are similarities between adults and pediatric results, it is still unclear if (patho-)physiological mechanisms differ concerning glucagon and glycemic state. This is exemplified by a study (Kahn et al.) (34) that grouped youths and adults with IGT and T2D and reported hyperresponsive beta cells, lower insulin sensitivity but no elevated secretion of glucagon in young compared to adult patients. Authors concluded that alpha cell dysfunction does not explain the difference in beta cell function. Increased fasting glucagon could be found with decreasing hepatic insulin sensitivity and correlation between fasting glucagon and fasting glucose was positive in adults, while negative in adolescents (34). In our study, there was an inverse fasting relationship in adolescents with IGT and IGT+IFG, but a positive association between glucose and glucagon in patients with IFG or T2D. The clear to strong correlation indicating a role for glucagon in pediatric T2D (34). As both, Kahn et al. and this study, are cross-sectional studies they do not allow for a causal inference and call for longitudinal studies.

Our study has several weaknesses we would like to address. The sample size, especially in T2D, is small, hence only descriptive analysis was possible for the six group observations. MRI data was only available in a limited number of patients. Individuals were of Caucasian ethnicity only and results may only be valid for Caucasian adolescents. As this was a cross sectional study design we do not have any follow-up data and cannot answer questions concerning specific mechanisms of causality. Glucagon response in OGTT differs from a mixed meal test (MMT) and it would have been of interest to study differences between OGTT and MMT in adolescents (27). Strengths of this study are the balance between sexes at an almost 50/50 ratio, the samples during OGTT being drawn at short intervals hence providing a closer insight, especially into early dynamics. To our knowledge this is the first study to look into glucagon dynamics in adolescents stratified by glycemic state (NG, IGT, IFG, IGT+IFG, T2D) as well as normoglycemic individuals with obesity or overweight and has normal weight controls. The size of the study population also exceeds those in prior studies pediatric studies on glucagon dynamics. Despite this, further subgrouping of the six subgroups was not possible due to sample size. Several gaps of knowledge remain. In general, more (longitudinal and modeling) studies in the pediatric population concerning glucagon and its role in metabolic disease are needed. What are the differences in mechanisms and progression of metabolic pathophysiology between pediatric and adult populations? Does hyperglucagonemia in younger children have different effects than in adolescents?

In conclusion, fasting and dynamic glucagon levels are elevated in young individuals with overweight or obesity and IFG compared to normoglycemic individuals with normal weight. They are, however, lower than in IGT, IFG+IGT and T2D. In subjects with IFG, glucagon showed a weak correlation with plasma glucose levels in the fasting state and during the first hour of OGTT. This is in line with adult data, indicating maintained normal insulin action in IFG but not IGT and hence lower glucagon secretory levels. All glycemic groups showed an overall suppression of glucagon during OGTT albeit starting at a higher glucagon concentration than in NW subjects. An initial increase of glucagon within the first 15 minutes could be observed in individuals with T2D. Glucagon in adolescents, as in adults, may play a role in the disease progression of T2D and further research in the pediatric population is necessary.

## Data availability statement

The raw data supporting the conclusions of this article will be made available by the authors, without undue reservation.

## Ethics statement

The studies involving humans were approved by Uppsala regional ethical committee (Number 2012/318) and the Salzburg ethical committee (Number 1544/2012). The studies were conducted in accordance with the local legislation and institutional requirements. Written informed consent for participation in this study was provided by the participants’ legal guardians/next of kin.

## Author contributions

TP: Conceptualization, Visualization, Writing – original draft, Writing – review & editing. TC: Conceptualization, Data curation, Writing – review & editing. WL: Data curation, Formal analysis, Investigation, Methodology, Writing – review & editing. GZ: Formal analysis, Writing – review & editing. KMö: Data curation, Investigation, Methodology, Writing – review & editing. JL: Writing – review & editing. DF: Writing – review & editing. EA: Writing – review & editing. SG: Data curation, Investigation, Project administration, Writing – review & editing. KMa: Data curation, Writing – review & editing. AF: Conceptualization, Writing – review & editing. CA: Writing – review & editing. JC: Formal analysis, Investigation, Validation, Writing – review & editing. DW: Conceptualization, Data curation, Investigation, Methodology, Project administration, Resources, Supervision, Writing – original draft, Writing – review & editing. PB: Writing – review & editing.
